# Low-frequency and rare variants may contribute to elucidate the genetics of major depressive disorder

**DOI:** 10.1038/s41398-018-0117-7

**Published:** 2018-03-27

**Authors:** Chenglong Yu, Mauricio Arcos-Burgos, Bernhard T. Baune, Volker Arolt, Udo Dannlowski, Ma-Li Wong, Julio Licinio

**Affiliations:** 1 Centre for Population Health Research, School of Health Sciences and Sansom Institute of Health Research, University of South Australia, Adelaide, SA Australia; 2 Mind and Brain Theme, South Australian Health and Medical Research Institute, Adelaide, SA Australia; 3 College of Medicine and Public Health, Flinders University, Bedford Park, SA Australia; 4 GENIUROS group, Center for Research in Genetics and Genomics, Institute of Translational Medicine, School of Medicine and Health Sciences, Universidad del Rosario, Bogotá, Colombia; 5 Discipline of Psychiatry, Adelaide Medical School, University of Adelaide, Adelaide, SA Australia; 6 Department of Psychiatry and Psychotherapy, University of Münster, Münster, Germany; 7 Department of Psychiatry and Psychotherapy, University of Marburg, Marburg, Germany; 8 Departments of Psychiatry, Pharmacology and Medicine, College of Medicine, State University of New York, Upstate Medical University, Syracuse, NY USA

## Abstract

Major depressive disorder (MDD) is a common but serious psychiatric disorder with significant levels of morbidity and mortality. Recent genome-wide association studies (GWAS) on common variants increase our understanding of MDD; however, the underlying genetic basis remains largely unknown. Many studies have been proposed to explore the genetics of complex diseases from a viewpoint of the “missing heritability” by considering low-frequency and rare variants, copy-number variations, and other types of genetic variants. Here we developed a novel computational and statistical strategy to investigate the “missing heritability” of MDD. We applied Hamming distance on common, low-frequency, and rare single-nucleotide polymorphism (SNP) sets to measure genetic distance between two individuals, and then built the multi-dimensional scaling (MDS) pictures. Whole-exome genotyping data from a Los Angeles Mexican-American cohort (203 MDD and 196 controls) and a European-ancestry cohort (473 MDD and 497 controls) were examined using our proposed methodology. MDS plots showed very significant separations between MDD cases and healthy controls for low-frequency SNP set (*P* value < 2.2e−16) and rare SNP set (*P* value = 7.681e−12). Our results suggested that low-frequency and rare variants may play more significant roles in the genetics of MDD.

## Introduction

Major depressive disorder (MDD) is a common mental illness with tremendous medical, economic, and social impact. MDD, as a principal contributor to disease load worldwide, leads to high levels of morbidity and mortality^1–5^. One significant avenue for preventing and treating depression lies in uncovering the genetics of this condition^6,7^. Despite rapid advances on genome-wide association studies (GWAS)^8–10^, little is understood about its fundamental biological basis and much further research needs to be carried out to fully unravel the genetic elements that confer susceptibility to this disorder^11,12^.

Many studies have been proposed to explore the genetic causes of complex diseases from a point view of the “missing heritability”^13–16^. For example, some genetic effects are not owing to the common single-nucleotide polymorphisms (SNPs) examined in the candidate-gene studies or GWAS, but due to low-frequency and rare variants, copy-number variations, and other types of genetic mutations^17^. Actually, GWAS focus on the identification of significant common (minor allele frequency (MAF) ≥ 5%) variants, thus analyses of low-frequency (0.5% ≤ MAF < 5%) and rare (MAF < 0.5%) variants would be promising to elucidate additional disease risk or trait variability^18^. Furthermore, using next-generation sequencing, family-based linkage analysis has also provided an important way to understand the role of rare variants in disease etiology^19,20^. For example, some family-control studies applied Hamming distance to identify disease genes based on sequencing data^21,22^. However, high-priced sequencing expenses are currently a concern that restricts acquiring large datasets.

Recently, we have applied GWAS and rare-variant analysis to investigate the genetics of MDD based on a whole-exome genotyping data from a Mexican-American cohort in Los Angeles and a replication European-ancestry cohort^23^. Our results suggested that the “missing heritability” in MDD may be partly explained by rare variants, because most of the functional variations detected in the cohorts were rare. In this study, we designed a novel computational and statistical strategy to further investigate this conclusion. In our methodology, we used Hamming distance on common, low-frequency, and rare SNP sets to measure the genetic distance between two individuals. Then we built the multi-dimensional scaling pictures, in which separation between MDD cases and healthy controls revealed valuable information for hidden genetic factors of major depression. The corresponding statistical results in the pictures were reported.

## Materials and methods

### The two cohorts used in this study

In our previous work^23^, we have investigated a cohort of MDD cases (*n* = 203) and controls (*n* = 196) of Los Angeles Mexican-Americans. They were mostly recent immigrants born in Mexico and experienced high levels of hyperactivation of the hypothalamic-pituitary-adrenal axis related to distress, challenges, and acculturation issues caused by immigration. MDD were diagnosed using the Structured Clinical Interview for DSM-IV (Diagnostic and Statistical Manual IV edition) (SCID, for abbreviation). Subjects met the diagnostic criteria for current, unipolar major depressive episode, attended a pharmacogenetic study on antidepressant treatment, and had an initial 21-Item Hamilton Depression Rating Scale (HAM-D21 for abbreviation) score of ≥18 with item number 1 (depressed mood) rated ≥2. Controls responded that they were in good health and replied questionnaires about acculturation. But they were not screened for medical illnesses and did not respond to structured psychiatric interviews. The controls were also Mexican-American and recruited from the same community in Los Angeles. The control group have similar sex ratio and age distribution (mean and standard error) to the MDD group (see Table S1). Participants submitted written informed consent, and their demographic, epidemiological, and clinical descriptions were previously described in detail^24–26^. We have registered this study in ClinicalTrials.gov (NCT00265291). The research was approved by the Institutional Review Boards of the University of California Los Angeles and University of Miami, USA, and by the Human Research Ethics Committees of the Australian National University and Bellbery Ltd, Australia.

We also included the European-ancestry cohort of MDD cases (*n* = 473) and controls (*n* = 497), which was used for replication in our previous study^23^. In this cohort, the MDD group also have similar sex ratio and age distribution (mean and standard error) to the control group (see Table S1). Those participants provided written informed consent and were recruited under two protocols: (1) Münster mood disorder studies (consisted of the neuroimaging and the mood-in-flame studies), which have been conducted by the Department of Psychiatry and Psychotherapy, University of Münster, Münster, Germany, and (2) the Characteristics of the Cognitive Function and Mood Study (CoFaM-Study) conducted by the Discipline of Psychiatry, University of Adelaide, South Australia, Australia^27^. The SCID/MINI (Mini International Neuropsychiatric Interview) was used to ascertain that healthy controls were free from lifetime history of psychiatric disorders; for this cohort, we also used DSM-IV criteria and HAM-D21 for the main diagnostic of MDD and mood assessment. The study on this cohort was approved by Human Research Ethics Committee protocols at the University of Münster, Germany, and University of Adelaide and Flinders University, South Australia, Australia.

Our previous power analysis on the same cohorts^23^ suggested that, when 100,000 variants are tested for association studies, 200 cases and 200 controls are sufficient to detect 80% true positives and a medium size of effect defined by the Cohen’s *h* parameter. This value of 100,000 overcomes the numbers of SNPs in common, low-frequency and rare-variant groups studied here. Actually, based on the effect sizes for the 19 MDD GWAS significant variants in the Mexican-American cohort^23^, the post hoc statistical power ranges between >60% (SNP-exm 2249659, Cohen’s *h* = 0.335) and >99% (SNP-exm1508600, Cohen’s *h* = 0.643). Cohen’s *h* suggests 0.2, 0.5, and 0.8 represent small, medium, and large effect sizes, respectively; thus, it indicates that our study had enough power to detect medium to large effect sizes for current association tests.

### Whole-exome SNP genotyping

The two cohorts were genotyped by the Australian Genome Research Facility (North Melbourne, VIC, Australia; www.agrf.org.au) using the Illumina HumanExome BeadChip-12v1_A, in which exonic content consists of >250,000 markers representing diverse populations and a range of common conditions. All the human samples passed the Illumina expected SNP calling rate (>99%). Then we filtered the raw whole-exome SNPs by a pipeline considering variant call rate, allele numbers, and Hardy–Weinberg equilibrium deviations. For this follow-up study, we analyzed 83,898 SNPs for the Mexican-American cohort and 121,174 SNPs for the European-ancestry cohort, which remained after quality control (QC) and filtering out criteria. Detailed QC and filtering analyses have been well reported in our previous work^23^.

### SNP classification and population stratification

Considering MAF, we divided the 83,898 SNPs in the Mexican-American population into 27,575 common variants, 17,838 low-frequency variants, and 38,485 rare variants, and divided the 121,174 SNPs in the European-ancestry population into 12,530 common variants, 12,902 low-frequency variants, and 95,742 rare variants. As expected, we found that most SNPs are rare (MAF < 0.5%) in the HumanExome BeadChip, because this chip has been designed to concentrate on rare variants rather than common ones^28^, which contrast to conventional genome-wide genotyping arrays that do not tag low-frequency and rare variants^29^.

We then used the four classes of SNPs (all, common, low-frequency and rare) to check population stratifications of the two cohorts. PLINK software^30^, which provides a powerful tool for population stratification based on pairwise identity-by-state (IBS) distance and multi-dimensional scaling (MDS) plots, was used here.

### Hamming distance between two individuals

In this study, we use Hamming distance^31^, a natural distance without assuming any model mutation/substitution rate, to investigate the genetic distance between two individuals based on a set of SNPs.

Let *S* be an SNP set which contains *n* SNPs. We use $SNP_k$ to represent the SNP indexed *k* (*k* = 1, …, *n*). Thus, *S* = {$SNP_1,\,\,SNP_2,\,\,...,\,\,SNP_n$}. Suppose that *X* and *Y* are two individuals who have their own genotypes on this SNP set *S*, namely and respectively, *S*_*X*_ and *S*_*Y*_ . Let *S*_*X*_ be {$SNP_1^X,\,\,SNP_2^X,\,\,...,\,\,SNP_n^X$} and *S*_*Y*_ be {$SNP_1^Y,\,\,SNP_2^Y,\,\,...,\,\,SNP_n^Y$}. Then the Hamming distance between the two individuals *X* and *Y* is defined as

1 $$H(X,Y) = \mathop {\sum}\limits_{i = 1}^n {\delta (SNP_i^X,SNP_i^Y)},$$

where $$\delta (a,b) = \left\{ {\begin{array}{*{20}{l}} 0 \hfill & {{\mathrm{if}}{\kern 1pt} a\,{\mathrm{and}}\,b\,{\mathrm{are}}\,{\mathrm{the}}\,{\mathrm{same}}} \hfill \cr 1 \hfill & {\mathrm{otherwise}} \hfill \end{array}} \right.$$, that is, the number of positions at which the corresponding SNPs are different on the SNP set *S*. Considering the size of the SNP set, we can also get the normalized Hamming distance as

2 $$NH(X,Y) = \frac{{\mathop {\sum}\limits_{i = 1}^n {\delta (SNP_i^X,SNP_i^Y)} }}{n}.$$

Take Table 1 as a simple example, individuals *X*, *Y*, and *Z* show their genotypes on an SNP set of eight SNPs. The Hamming distance between *X* and *Y* is 7 (SNP1, SNP3, SNP4, SNP5, SNP6, SNP7, and SNP8 are different). Similarly, the Hamming distance between *Y* and *Z* is 3, and the Hamming distance between *X* and *Z* is 5. Our hypothesis was that if two individuals have closer Hamming distance in this way, then those two individuals would have closer genetic distance and more similar phenotypes such as diseases or traits. We assume that *Y* and *Z* have more similar phenotypes in the above example.

**Table 1:** Hamming distances of three subjects in an 8-SNP set

Genotype	SNP1	SNP2	SNP3	SNP4	SNP5	SNP6	SNP7	SNP8
	(A/T)	(G/T)	(C/G)	(C/T)	(C/T)	(A/G)	(A/C)	(C/T)
Subject *X*	AT	GG	CG	CC	CC	AG	AC	TT
Subject *Y*	AA	GG	CC	TT	CT	GG	CC	CT
Subject *Z*	AA	GG	CC	CC	CT	AA	CC	TT

SNP single-nucleotide polymorphism

Given a group of individuals, we can compute their Hamming distance matrix based on a specific SNP set such as common, low-frequency, or rare-variant set. After obtaining the distance matrix between all pairs of individuals, MDS approach^32^ can be used to observe the distance relationships among those individuals in a two-dimensional graph. The display of scatters representing individuals which shows separating variability between MDD cases and healthy controls can reveal interesting genetic information hidden in the SNP sets. Then for statistical analysis, Hotelling’s *T*^2^ test for two independent samples is used to examine whether the means of the two groups (case and control) are equal.

### Code availability

The codes (by R software; www.r-project.org) of data analysis for this study can be accessed from the authors.

## Results

### Population stratification

**Fig. 1:**
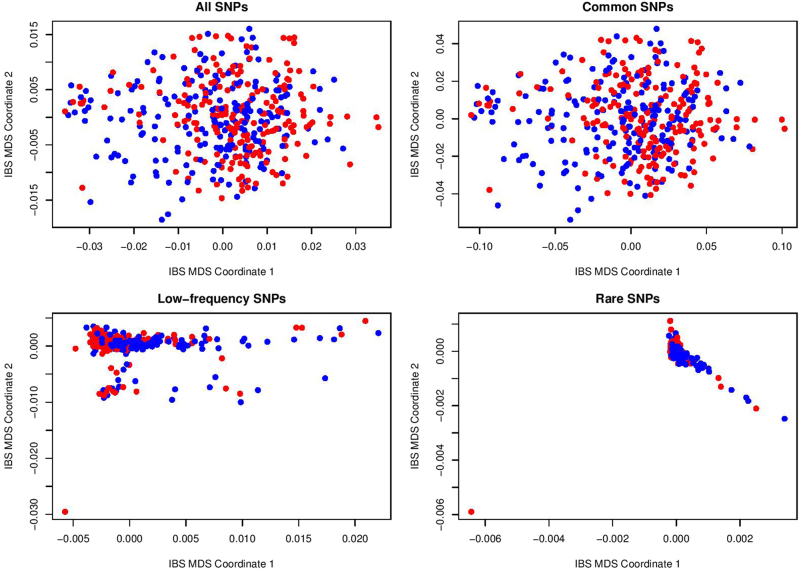
Population stratification based on IBS distance and MDS for Mexican-American cohort

**Fig. 2:**
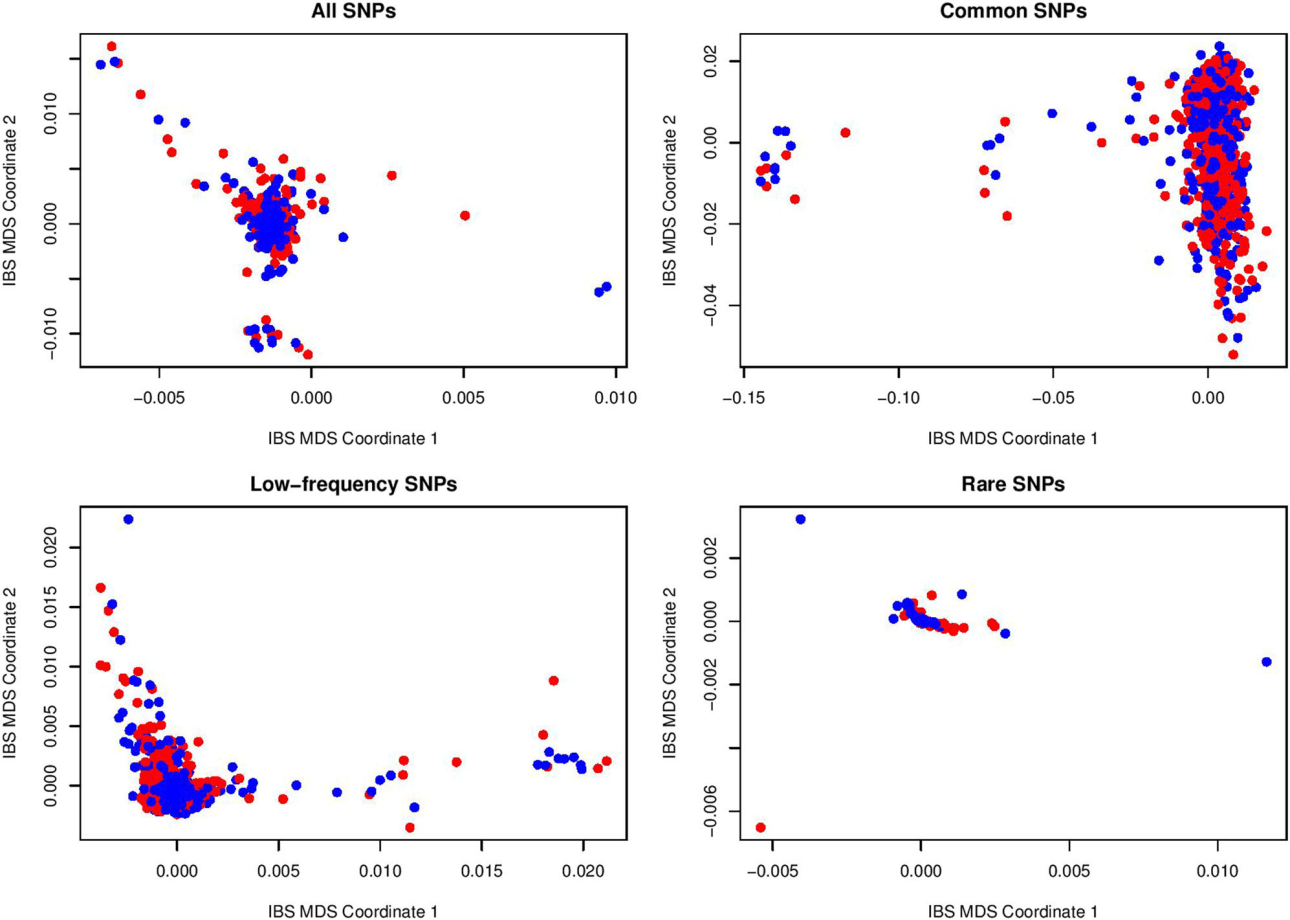
Population stratification based on IBS distance and MDS for European-ancestry cohort

In Figs. 1 and 2, we presented the population stratification results based on all, common, low-frequency, and rare SNP sets for the Mexican-American cohort and the European-ancestry cohort. Although several far outliers are found in Mexican-American cohort for low-frequency and rare SNPs and in European-ancestry cohort for rare SNPs, there is no significant separation between depressed cases (blue points) and controls (red points) in the IBS-MDS plots.

### MDS on Hamming distance

**Fig. 3:**
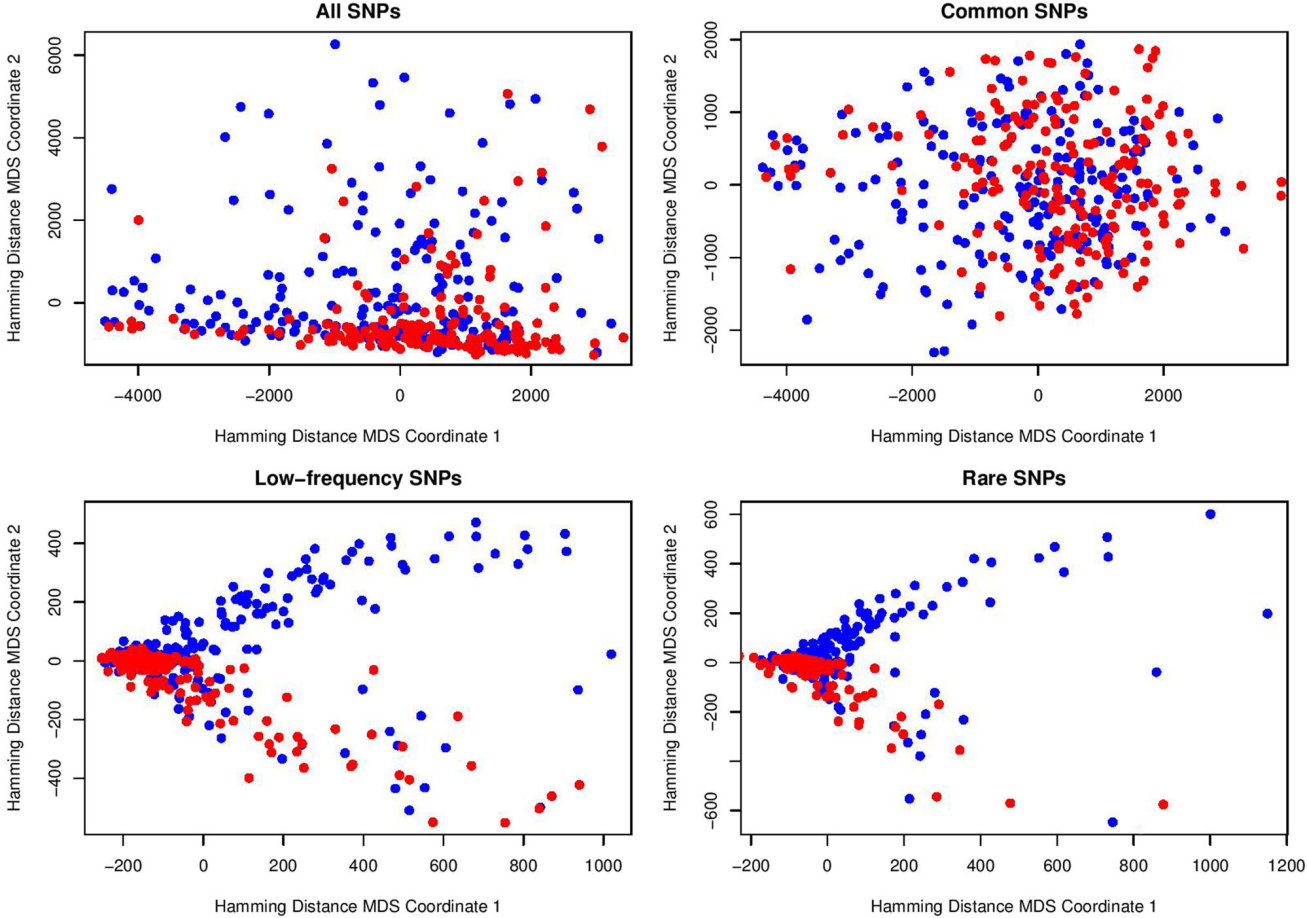
MDS visualization based on Hamming distance for Mexican-American cohort

In Fig. 3 we presented MDS results on Hamming distance for all, common, low-frequency, and rare SNP sets of the Mexican-American cohort. For common SNPs, there is no significant separation between MDD cases (blue points) and controls (red points). However, for low-frequency and rare SNPs, we found that all the healthy controls were scattered in the lower half-plane, and all points in the upper half-plane were blue points representing depressed cases. We use Hotelling’s *T*^2^ test to statistically examine these visual separations between case and control points in the MDS plane. For common variants, the result is *P* value = 5.891e−05 and *T*^2^ statistic = 9.983. For low-frequency variants, the Hotelling’s *T*^2^ test result is *P *value <2.2e−16 and *T*^2^ statistic = 42.958. For rare variants, the Hotelling’s *T*^2^ test result is *P *value = 7.681e−12 and *T*^2^ statistic = 27.32. Therefore, the separation of cases and controls shown in the MDS plane is much more significant for low-frequency and rare SNPs than for common SNPs.

**Fig. 4:**
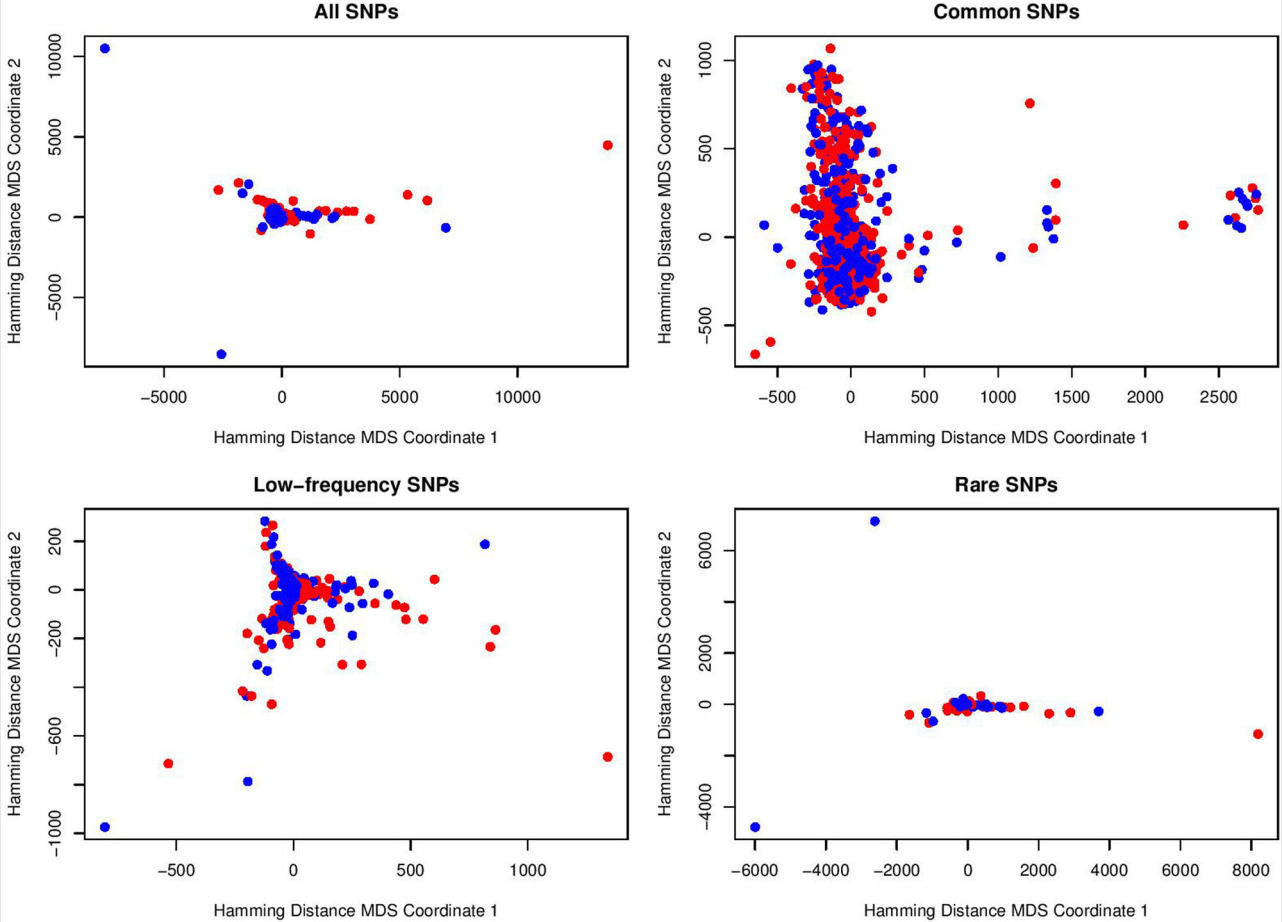
MDS visualization based on Hamming distance for European-ancestry cohort

In Fig. 4, we showed the results of MDS on Hamming distance for all, common, low-frequency, and rare SNP sets of the European-ancestry cohort. For all the SNP sets, we see that there is no significant visual separation between MDD cases (blue points) and controls (red points) in the MDS planes. The same results can also be found in Figure S1 by excluding some far outliers.

## Discussion

Genetic factors play important roles in the susceptibility to major depression, as indicated by family, twin, and adoption studies^33^. The heritability of MDD is estimated to range between 40 and 70%^34^. In this article, we designed a novel methodology to explore the “missing heritability” of MDD. The significant separations between cases and controls for low-frequency and rare variants in the planes of MDS on Hamming distance supported our previous conclusion^23^ that most of the functional variants detected in the Mexican-American cohort were rare. The corresponding statistical results also show that the separation for low-frequency and rare SNPs is much more significant than for common SNPs. Thus, our findings further suggest that low-frequency and rare variants may play more significant roles in the development of MDD. Low-frequency and rare variants are currently not tagged by conventional genome-wide genotyping arrays, thus they may represent an important but understudied component of MDD genetics^29^. There are many different types of technical designs that identify low-frequency and rare variants. In the current study, we applied whole-exome-wide genotyping array data, which are relatively cost-effective. With the rapid development of next-generation sequencing technologies (whole-genome sequencing, whole-exome sequencing, and targeted sequencing of candidate genes), it is now possible to collect most or even all low-frequency and rare genetic variants in large samples and test their roles in human disease risk^35–37^. Thus, future work would be needed to further investigate our methodology on much larger SNP sets.

The traditional genetic distance^38^ considers mutation rates and is designed as a measure of genetic divergence between populations within a species. Therefore, it is not appropriate to examine the genetic variation associated with a complex disorder within a human population, namely Mexican-American. In this work, Hamming distance was used to investigate the genetic distance between two individuals based on their SNP sets. As sequencing costs are currently dropping further, we may examine single-nucleotide variants (SNVs) which involve much more individual genetic information. For example, we recently proposed a new concept of SNV proportion in genes and employed it to develop a predictive approach for major depression^39^. Using similar classification and cluster analysis methods, a potential tool for MDD diagnosis could also be constructed based on Hamming distance and low-frequency/rare SNPs.

Using our methodology with a case–control study, we could examine the effect of a group of variants within a specific range of MAF. The design and methodology we have developed can also be extended to other complex disorders. Our approach based on exome genotyping array or sequencing data may reveal the “missing heritability” resulted from allele frequency for many complex diseases.

Our European-ancestry cohort failed to show significant separations of cases and controls in the MDS planes on Hamming distance. The reasons could be as follows. First, MDD is clearly a gene–environment interaction disorder^34^. Our Mexican-American cohort is comprised of first-generation individuals (60%) who have experienced significant levels of stress and hyperactivation of the hypothalamic-pituitary-adrenal axis related to acculturation issues^40,41^. In contrast to the European-ancestry cohort, the significant stressful life events for Mexican-Americans could cause much higher levels of depression. Therefore, the depression effect differences between case and control in two cohorts may be large. Further studies using our methodology could be tested on a larger size of European-ancestry sample. Second, the European-ancestry cohort that we studied have much lower levels of genetic variants. In our previous work^23^, whole-genome sequencing analyses of a subset of the two cohorts revealed that European-ancestry subjects have a significantly reduced (around 50%) number of SNVs compared with Mexican-American subjects. For this reason, the roles of low-frequency and rare variants may vary across populations.

## Electronic supplementary material

Supplementary Information(PDF 263 kb)
