# Supplementary material for: Low-frequency and rare variants may contribute to elucidate the genetics of major depressive disorder
Source: Transl Psychiatry. 2018 Mar 27;8:70. doi: 10.1038/s41398-018-0117-7 (PMC5913271; doi:10.1038/s41398-018-0117-7)

# Low-frequency and rare variants may contribute to elucidate the genetics of major depressive disorder

Supplementary Information

Chenglong Yu, Mauricio Arcos-Burgos, Bernhard T. Baune, Volker Arolt, Udo Dannlowski, Ma-Li Wong, Julio Licinio

This supplementary information includes Table S1 and Figure S1.

**Table S1**. Breakdown of descriptive statistics for MDD cases and controls. Std Err, standard error.

| Cohort | Status | Sex | Number | Age Mean | Age Std Err |
|---|---|---|---|---|---|
| Mexican-American | MDD | Male | 70 | 40.96 | 1.45 |
| | | Female | 133 | 39.81 | 1.05 |
| | Control | Male | 80 | 39.34 | 1.36 |
| | | Female | 116 | 39.03 | 1.13 |
| European-Ancestry | MDD | Male | 216 | 38.15 | 0.94 |
| | | Female | 257 | 39.30 | 0.79 |
| | Control | Male | 211 | 32.63 | 0.78 |
| | | Female | 286 | 32.41 | 0.73 |

**Figure S1**. Multi-Dimensional Scaling (MDS) visualization based on Hamming distance for European-ancestry cohort without far outliers.

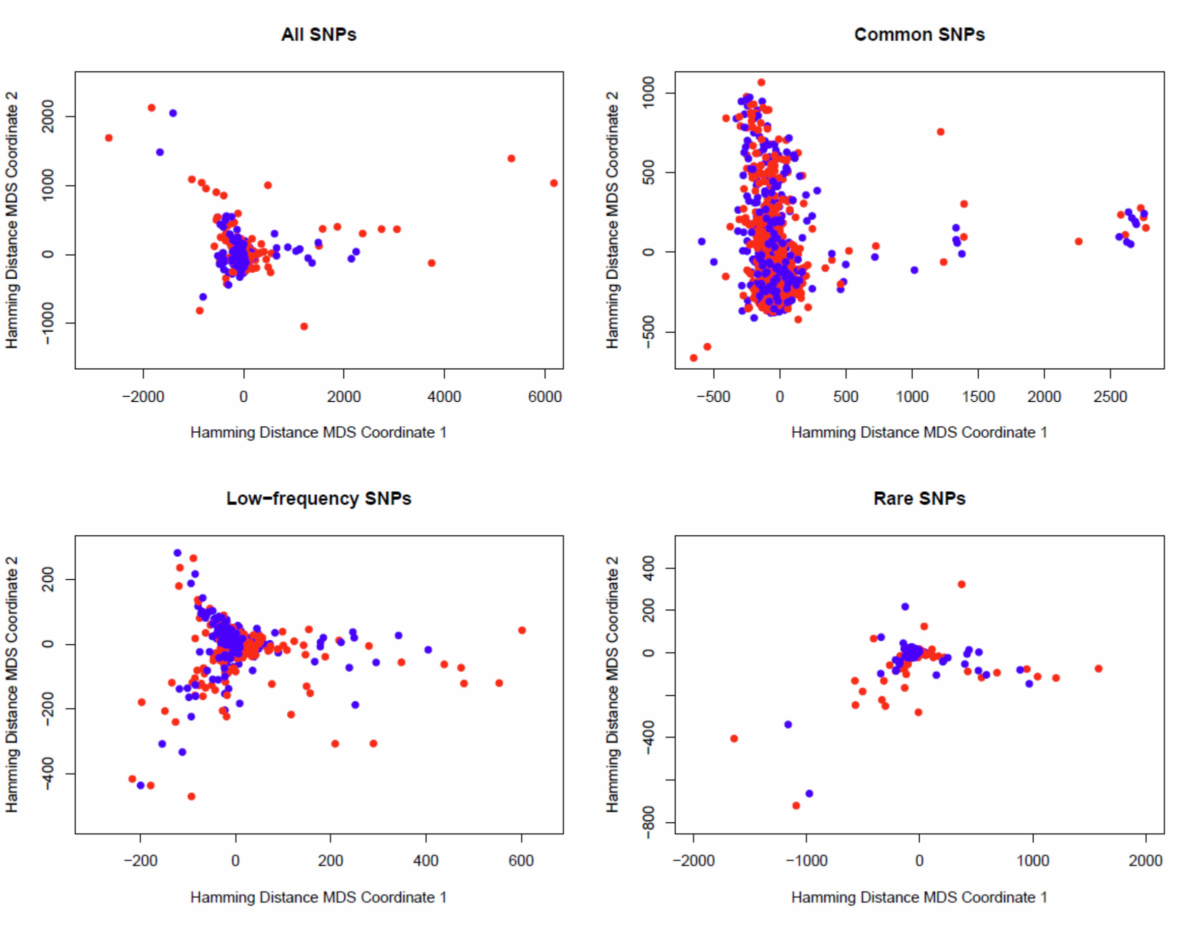

Supplement: Supplementary file 1 — Supplementary Information(PDF 263 kb) [file 41398_2018_117_MOESM1_ESM.pdf]
